# Supplementary material for: First Detection of Hepatitis E Virus (Rocahepevirus ratti Genotype C1) in Synanthropic Norway Rats (Rattus norvegicus) in Romania
Source: Viruses. 2023 Jun 7;15(6):1337. doi: 10.3390/v15061337 (PMC10302290; doi:10.3390/v15061337)
Supplement: Supplementary file 1 [file viruses-15-01337-s001.zip › Supplementary Figure S1.pdf]

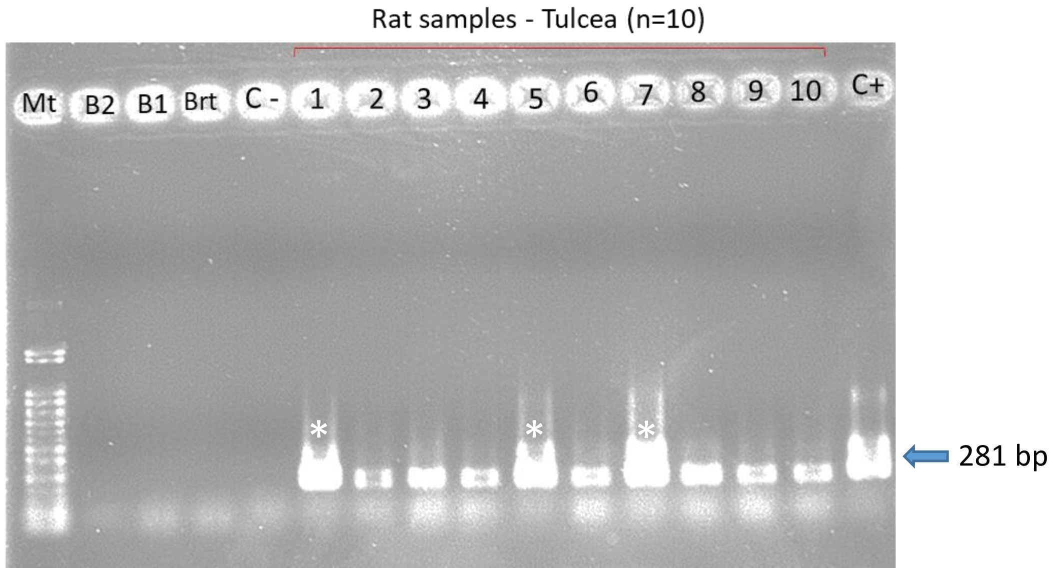

Supplementary Figure S1. Analysis of the results obtained by broad-spectrum RT-PCR for 10 samples collected from rats in Tulcea County (Mt, DNA size markers (DNA Molecular Weight Marker XIV- Roche); B2, B1, Brt, negative controls - free water, used in each PCR reaction round (RT, first PCR and nested PCR); C+, positive control. Expected product size is 281 bp. *Positive samples used for sequencing.
