# Supplementary material for: First Detection of Hepatitis E Virus (Rocahepevirus ratti Genotype C1) in Synanthropic Norway Rats (Rattus norvegicus) in Romania
Source: Viruses. 2023 Jun 7;15(6):1337. doi: 10.3390/v15061337 (PMC10302290; doi:10.3390/v15061337)
Supplement: Supplementary file 1 [file viruses-15-01337-s001.zip › Supplementary Table S1.pdf]

Supplementary Table S1. HEV genotypes targeted and primers used for each RT-PCR assay

Genotype	Assay	Region	Position	Step	Primers /Probes	Sequence (5'-3')	Reference
HEV-1 to HEV-4 genotypes	TaqMan real-time RT-PCR	ORF3	5261–5330 based on GenBank accession no. M73218	RT-qPCR	JVHEV-R	AGGGGTTGGTTGGATGAA	Jothikumar et al., 2006 [35]
					JVHEV-F	GGTGGTTTCTGGGGTGAC	
					TaqMan probe	TGATTCTCAGCCCTTCGC	
	RT-nested PCR	ORF2	5996 to 6343 based on GenBank accession no. M73218	First PCR	3156N	AATTATGCC(T)CAGTAC(T)CGG(A)GTTG	Cooper at al., 2005 [37]
					3157N	CCCTTA(G)TCC(T)TGCTGA(C)GCATTCTC	
				Nested PCR	3158N	GTT(A)ATGCTT(C)TGCATA(T)CATGGCT	
					3159N	AGCCGACGAAATCAATTCTGTC	
Hepeviruses, including rat HEV.	NBS-RT-PCR	ORF1	4107–4387 based on GenBank accession no. GU345042	First PCR	HEV-cs	TCGCGCATCACMTTYTTCCARAA	Johne at al., 2010 [39]
					HEV-cas	GCCATGTTCCAGACDGTRTTCCA	
				Nested PCR	HEV-csn	TGTGCTCTGTTTGGCCCNTGGTTYCDG	
					HEV-casn	CCAGGCTCACCRGARTGYTTCTTCCA	
